# Frequency of ICD-10 psychiatric diagnosis in children with intellectual disability in Lahore, Pakistan & Caregivers Perspective

**DOI:** 10.12669/pjms.312.7319

**Published:** 2015

**Authors:** Nazish Imran, Muhammad Waqar Azeem, Ahsan Sattar, Mohammad Riaz Bhatti

**Affiliations:** 1 Dr. Nazish Imran, MBBS; MRC Psych (London), Chairperson & Head, Associate Professor, Department of Child & Family Psychiatry, King Edward Medical University/Mayo Hospital, Lahore, Pakistan; 2 Dr. Muhammad Waqar Azeem, MD, FAACAP, DFAPA, Medical Director & Chief of Psychiatry, Albert J. Solnit Children’s Center, Associate Clinical Professor Yale Child Study Center, Yale University School of Medicine, Middletown, USA; 3 Ahsan Sattar, MSc (Psychology), Department of Child & Family Psychiatry, King Edward Medical University/Mayo Hospital, Lahore, Pakistan; 4 Muhammad R. Bhatti, F.R.C. Psych., M.R.C. Psych., Former Professor, Academic Department of Psychiatry & Behavioural Sciences, King Edward Medical University, Lahore, Pakistan

**Keywords:** Children and adolescents, Intellectual disability, Psychopathology, Pakistan

## Abstract

**Objective:**

Association between Intellectual disability (ID) and psychiatric disorders in children & adolescents is well established but there is a paucity of published studies from Pakistan on this topic. The main aim of the study was to assess the frequency of ICD-10 psychiatric diagnosis in the hospital outpatient sample of children with ID in Lahore, Pakistan as well as to find out which challenging behaviors, caregivers find difficult to manage in this setup.

**Methods:**

Socio-demographic information was collected, Wechsler Intelligence Scale for Children-Revised & ICD-10 diagnostic criteria was used to assess children (age range 6 – 16 years) with suspected ID along with identification of behaviors found to be difficult to manage by caregivers.

**Results:**

150 children were assessed with mean age of 10.7 years (males 70 %). Majority (72%) had mild ID while 18.7% and 9.3% had moderate and severe ID respectively. Thirty percent of children met the criteria for any psychiatric diagnosis, the most common being Oppositional Defiant Disorder (14%) and Hyperkinetic Disorders (10%). Verbal and physical aggression, school difficulties, socialization problems, inappropriate behaviors (e.g. disinhibition), sleep & feeding difficulties were the significant areas identified by the caregivers as a cause of major concern.

**Conclusions:**

Significantly high prevalence of ICD-10 psychiatric diagnosis in children with ID was found in Lahore, Pakistan. Support services for these children should be responsive not only to the needs of the child, but also to the needs of the family.

## INTRODUCTION

Children & Adolescents with Intellectual Disability (ID) have high rates of mental health problems and behavioral difficulties. Various prevalence rates for psychopathology in ID have been reported, for example 50% in sample of children with severe mental retardation in classical Isle of Wight study.^1^ Other studies reported prevalence in various samples ranging from 31% to 47%.^2^-^6^ All these studies highlighted that children with ID are at significantly higher risk for psychopathology as compared with their non-ID peers. Poor communication, sensory disabilities, epilepsy, physical illness, medications, limited coping strategies have been identified as some of the predisposing factors for mental health problems in this group.^7^ These children with various accompanying diagnosis poses many diagnostic and therapeutic challenges as the needs for adequate mental health services for these children largely remains unmet. These psychiatric disorders can further lead to deterioration in the functioning capacity of these children.

Pakistan has one of the highest reported prevalence of Intellectual disability in the world. In a sample of 6365 children in Pakistan, reported rates of mild mental retardation was 6.5% and severe cognitive disability was 1.9%.^8^,^9^ Another study from Karachi, Pakistan reported estimated rates of mental retardation and learning disability as being 19.0/1000.^10^ Mubbashar et al. reported rates of severe mental retardation being 16/1000 in a sample of 3 to 9 years old children.^11^ Clinical experience suggests that a significant proportion of these children with ID presents with psychiatric and behavioural difficulties, which are not only difficult to manage but are also associated with extreme burden of care for the families. To compound the problem, psychiatric disorders in these children are often, not adequately identified because of insufficient number of psychiatrists with experience in this field.

Although the evidence for association between ID and psychiatric disorders in children & adolescents is compelling, there is a paucity of published studies from Pakistan on this topic. The present study seeks to address this knowledge gap in identification of psychiatric disorders in children with ID by assessing the frequency of ICD-10 psychiatric diagnosis in the hospital outpatient sample of children with ID in Lahore, Pakistan. This study also looked at which behavioural issues, caregivers find difficult to manage in this setup to help identify service needs.

## METHODS

The study was approved by Institutional Review Board of King Edward Medical University, Lahore, Pakistan. Following informed consent from the parents, children between the ages of 6-16 years presenting to the outpatient of Child & Family Psychiatry Department, Mayo Hospital, Lahore with suspected ID were recruited in the study. A structured questionnaire was used to collect relevant demographic and family information. IQ was assessed by Wechsler Intelligence Scale for Children-Revised (WISC-R). ICD-10 diagnostic criteria were used to diagnose various psychiatric disorders. Information was also collected to determine areas which were reported by parents to be difficult to manage among these children and considered as a cause of concern.

The data was analyzed by using SPSS version 17.0. Descriptive statistics were determined for various factors. Association of level of ID with various psychiatric diagnoses, and of different diagnosis with areas of concern for parents was determined by using Chi- square tests. For all purposes, a P-value of <0.05 was considered statistically significant.

## RESULTS

Main informant were mothers in 70% of children. More than half of informants (54%) had monthly income of less than 10,000 Pakistani Rupees (equal to about 100 US dollars). 57% lived in joint family setup. About two third of the informants had education up to tenth grade. Most of the parents denied any family history of psychiatric illness (90%) or intellectual disability (96%). Thirteen parents were under psychiatric care with diagnosis of depression (3.3%); epilepsy (2.7%); schizophrenia (1.3%) and two did not knew their diagnosis. Table-I gives the socio-demographic information of the study sample as well as their IQ levels.

**Table-I:** Socio- demographic information of children with ID (N=150).

	N (%)
Age (Mean; (s.d)	10.73(2.9)
Gender	
Male	105(70.0)
Female	45(30.0)
*Schooling*	
Not Attending	23(15.3)
Attending School	127(84.6)
Regular School	120(93.8)
Special School	6(4.7)
Madrassa	1(1.6)
History of Perinatal Asphyxia Present	45(30.0)
*History of*	
Epilepsy	17(11.3)
Physical Illness	6(4.0)
Hearing Impairment	3(2)
Speech Impairment	53(35.3)
*IQ Level*	
F-70Mild ID (50-69)	108(72.0)
F-71Moderate ID (35-49)	28(18.7)
F-72Severe ID (<35)	14(9.3)

Table-II gives the frequency of various ICD-10 psychiatric diagnoses in the sample. Around 70% of the sample did not meet the criteria for any ICD 10 diagnosis. Oppositional Defiant Disorder (F-91.3) and Hyperkinetic Disorders (F-90) were the most frequent disorders observed. We did not observe any statistically significant differences in ICD-10 Psychiatric diagnosis in relation to gender or IQ level in our sample.

**Table-II:** Frequency of ICD-10 Psychiatric Diagnosis in Children with Intellectual disability.

ICD-10 Psychiatric Diagnosis in Children	Sales
No Psychiatric Diagnosis	70%
F91.3 ODD	14%
F-90 Hyperkinetic Disorder	10%
F-23 Acute & Transient Psychotic Disorder	3%
F91.1 Unsocialized conduct disorder	1%
F84.0 Childhood Autism	1%
F-41.1 Gen- Anxiety Disorder	1%

Table-III gives details about the challenging behaviors, which were considered areas of concern by the caregivers of children with ID. Statistically significant differences were observed between various psychiatric diagnosis and areas of concern reported by parents. Children with ODD were more likely to have schooling problems and inappropriate behaviors while children with Hyperkinetic Disorders were more likely to have socialization difficulties (P value <.05). Children with autism were more likely to have self-injurious behaviour, feeding problems, inappropriate behaviors and socialization difficulties. Self-care, and inappropriate behaviors were more cause of concern for caregivers of children who had psychosis along with ID.

**Table-III:** Areas of concern reported by caregivers.

	Not at all N(%)	Somewhat true N(%)	Definitely a problem N(%)
Verbal Aggression	33(22%)	86(57.3)	31(20.7)
Physical aggression	70(46.7)	61(40.7)	19(12.7)
Self-injurious behavior	127(84.7)	20(13.3)	3(2.0)
Sleep difficulties	132(88)	15(10)	3(2.0)
Feeding difficulties	134(89.3)	14(9.3)	1(0.7)
Self-care	111(74.0)	36(24.0)	3(2.0)
Inappropriate behavior	70(46.7)	69(46.0)	11(7.3)
socialization	68(45.3)	72(48.0)	10(6.7)
Schooling problems	45(30.0)	63(42.0)	42(28.0)

## DISCUSSION

The study showed a high proportion of psychiatric diagnosis (30%) in our sample of children with ID, the results being consistent with previous literature reporting variable but consistently higher prevalence of psychopathology in this group.^1^,^12^-^15^ The variability may be because of different characteristics of sample like age, IQ 1evel, different types of population studied, different measures of psychopathology used as well as changes in diagnostic systems over the years. Overall studies have concluded that children with ID have at least three fold increased risk of significant psychopathology in comparison to children without ID.^14^ These results are of concern to health professionals as psychiatric problems impact negatively on life opportunities as well as social inclusion of children with ID.^16^

We found ODD & Hyperkinetic Disorders to be the most common psychiatric disorders in our sample. Hyperkinesis & any conduct disorder accounted for approximately 2/3^rd^ of all diagnosis among children with ID in Britian.^6^ Another study has shown very high rates of conduct disorder (45%) and depression (22%) among children with mild ID presenting to outpatient for treatment.^17^ Autism was diagnosed in only 1% of our sample which deviates from previous studies reporting much higher prevalence of Autism spectrum disorder.^5^ One possibility of this may be of our sample mostly comprising of children suffering from mild to moderate ID as prevalence of Autism increases with severity of ID. Another possibility may be the overlapping of symptoms of ID with Autism leading to underestimation of frequency of Autism in our sample, or high presence of autistic traits rather than children meeting full diagnostic criteria for Autism. There is also reluctance among health professionals in diagnosing Autism due to social stigma as well as lack of training in this field. Presence of these psychiatric disorders further decrease the functioning capacity of these children. To further compound the issue, there are huge hurdles in meeting their mental health needs because of lack of specialized services for children with ID. Literature suggests that psychiatric disorders may manifest differently in children and adolescents with ID and as ID becomes severe, accurate psychiatric diagnosis becomes more difficult.^18^ In the presence of very few health professionals with experience of diagnosing and managing these children; many psychiatric disorders remain undiagnosed and thus untreated; negatively impacting the child and family.

Parents of children with ID experience many challenging behaviors which are of extreme stress for the whole family.^19^-^21^ Our results also suggests that while addressing mental health needs of children with ID, special emphasis needs to be placed on addressing challenging behaviors like aggression, self-injury, schooling difficulties, social issues, sleep problems to name few which are of concern to the caregivers and difficult to manage. These results are in line with previous literature focusing on challenging behaviors in children with ID. Social problems, attention difficulties and aggression were the most prominent behavioral problems among children in a study comparing children with and without ID.^14^ Challenging behaviors may be the presentation of comorbid psychiatric disorder; whereas on the other hand, it may exacerbate the psychiatric disorder.^22^ In addition, learned behavior, environmental and social factors play an important role for these children and families.^23^ Parental stress and health outcomes has been found to be associated with child characteristics including age of the child, severity of ID, and coexisting behavior issues.^24^,^25^ Another important finding in our study was school related difficulties being a major concern despite the fact that most of the children were attending school. This may be explained by number of factors including limited number of special schools; stigma and reluctance of parents to send their child to special schools. In many instances, children get enrolled in regular schools which fail to cater for the special needs of these children. As a result they unfortunately continue to struggle and this becomes a major source of stress for both children and families.

Results of our study needs to be seen in the context of its limitations, which includes small sample size, lack of comparison group and inclusion of only hospital outpatient population. Future studies are needed which should include special needs schools in addition to the hospitals in multiple sites across the country; encompassing both urban and rural settings with a larger sample size.

In conclusion, we found a high prevalence of psychiatric diagnosis in children with ID. The study has clinical implications and call for continued education and awareness regarding the mental health needs of children with ID. This also emphasize that support services should be responsive not only to the needs of the child, but also to the needs of the family in which they are living.
